# An Optical Frequency Domain Reflectometer’s (OFDR) Performance Improvement via Empirical Mode Decomposition (EMD) and Frequency Filtration for Smart Sensing

**DOI:** 10.3390/s24041253

**Published:** 2024-02-15

**Authors:** Maxim E. Belokrylov, Dmitry A. Kambur, Yuri A. Konstantinov, D Claude, Fedor L. Barkov

**Affiliations:** 1 Perm Federal Research Center, Ural Branch of the Russian Academy of Sciences, 13a Lenin Street, 614990 Perm, Russia; belokrylovme@gmail.com (M.E.B.); kambur.dima@gmail.com (D.A.K.); cdf750@yandex.ru (D.C.); fbarkov@pstu.ru (F.L.B.); 2 Applied Mathematics Department, Perm National Research Polytechnic University, Komsomolsky Avenue 29, 614990 Perm, Russia

**Keywords:** optical frequency domain reflectometry, OFDR, optical measurements, empirical mode decomposition, auxiliary interferometer, gas cell

## Abstract

We describe a method for reducing the cost of optical frequency domain reflectometer (OFDR) hardware by replacing two reference channels, including an auxiliary interferometer and a gas cell, with a single channel. To extract useful information, digital signal processing methods were used: digital frequency filtering, as well as empirical mode decomposition. It is shown that the presented method helps to avoid the use of an unnecessary analog-to-digital converter and photodetector, while the OFDR trace is restored by the equal frequency resampling (EFR) algorithm without loss of high resolution and with good measurement repeatability.

## 1. Introduction

The development of smart cities is a process aimed at creating and improving the city’s infrastructure, which can better the quality of life of its residents, increase the efficiency of city services, and refine the environmental situation. Smart cities use state-of-the-art technologies such as the Internet of things [1], artificial intelligence [2], big data [3] to manage city systems, and improve the quality of life of citizens. As a result of this development, cities become safer, more comfortable, and environmentally friendly.

A smart city sensor system is a set of sensors and devices that collect information about the state of the environment and transmit it to a central computer or control system. In a smart city, a sensing system can be used to monitor and control various parameters, such as temperature [4], humidity [5], noise level [6], air quality [7], traffic, traffic jams, etc. [8,9,10]. This allows one to automatically regulate the operation of smart city systems, for example, turn on heating when the temperature drops, regulate the speed of transport depending on road congestion, etc. The sensing system can also be used to collect data on the behavior of citizens and their preferences, which helps improve the quality of life of city residents.

Sensors can be conditionally classified as pointwise sensors (where the sensing elements of the system are separate parts) and as distributed ones, where the study of physical characteristics is carried out along the entire length of the optical sensor. Optical fibers are mainly used as such a sensor. Distributed sensing systems make it possible to obtain more data from the object under study while using only one extended sensor.

The use of distributed fiber optic sensors (DFOSs) [11,12,13,14] for monitoring systems makes it possible to obtain information about the state of an object with high accuracy and reliability. They can be used to measure temperature [15], pressure [16,17], gas concentration [18], vibration [19,20], deformation [21,22], and other parameters. One of the main advantages of DFOSs is their high measurement resolution. They allow changes in parameters with a resolution of up to several tens of micrometers. This makes it possible to use DFOSs for monitoring the state of objects where high measurement precision is required, for example, in technology, aviation, or industry. Another advantage of DFOSs is their reliability. They do not require labor-intensive, frequent maintenance and have a long service life. In addition, they are not susceptible to electromagnetic interference.

**Figure 1:**
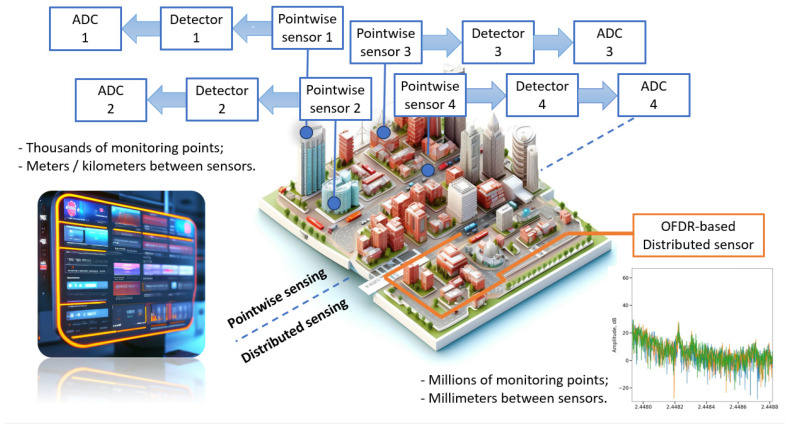
Smart city monitoring concepts: pointwise and distributed ones.

A significant part of the sensors that allow recording physical quantities with high spatial resolution are based on the principles of optical frequency domain reflectometry (OFDR) [23,24,25]. Such systems could become a good substitution for the pointwise sensors in smart city concept (Figure 1). Optical reflectometers designed to study back reflections in integrated optical chips and photonic integrated circuits are also based on the same principle. Typically, their transceiver modules consist of a wavelength-scanning highly coherent radiation source and a Mach–Zehnder or Michelson interferometer.

Obtaining millimeter precision and high repeatability of measurements along the spatial coordinate is possible both by maintaining the linearity of frequency scanning of a narrow-band laser source and by controlling the sweeping function and algorithmically correcting the data received from the sensor. The first approach can be implemented using a self-scanning fiber laser [26,27]. It adjusts the frequency by a fixed value within a time period strictly determined by the configuration of the laser circuit, so the data obtained by probing a fiber or integrated optical circuit with such a laser does not require serious additional processing. Unfortunately, the widespread use of such systems is currently hampered by the inability of such lasers to undergo wide (tens of nm) tunability in the wavelength region of 1.55 μm. That is why most modern researchers and developers choose the second path—compensation for nonlinearity using signal processing. To obtain the data necessary to compensate the nonlinearity, the basic reflectometer setup is supplemented with two additional reference channels. The first reference channel is a fiber Mach–Zehnder interferometer, where two parts of the same signal interfere, with one of them delayed in time using a delay line. A photodetector that registers the signal of a given reference channel, with absolutely linear frequency scanning, must produce a harmonic function with a linearly varying beat frequency. When the scanning speed changes, the beat frequency also changes:

(1) $$f_{beat}=\frac{2nL\gamma }{c}$$

where *L* is the length of the delay line, and *γ* is the tuning speed.

Using data from this reference channel makes it possible to compensate for the nonlinearity of laser scanning and improve the spatial resolution of the source data by several orders of magnitude. However, practice shows that to ensure good repeatability of measurements, it is necessary to accurately record not only the scanning speed, but also the frequency range of laser sweeping. For this purpose, a second reference channel is usually designed, into which a wavelength (frequency) reference is built. Ideally, it includes a gas cell, the composition of the contents of which is determined by the scanning range of the laser. Some studies use fiber Bragg gratings (FBGs) or Fabry–Perot interferometers (FPIs) for this purpose. However, the gas cell unit is more preferable due to the high temperature sensitivity of FBG and FPI. One way or another, the two reference channels have two independent detectors and analog-to-digital converters (ADCs). Although the main cost of a frequency domain reflectometer is usually the laser source, in OFDR systems that measure fiber lines tens of kilometers long with sufficiently high resolution, the cost of detectors and analog-to-digital converters becomes significant.

In [28], a method was proposed to get rid of one of the reference channels by including an additional filter in the line interrogated by a self-scanning laser. However, in some cases, this can reduce the dynamic range of the system, and its artificial increase by erbium amplifiers can lead to distortion of the output signal shape [29].

The number of channels in an OFDR system can be reduced not only by using a self-scanning laser. Thus, in [30], the fiber under test is used both to measure and to compensate for the nonlinearity of laser frequency sweeping, which allows the system to level out phase errors without an auxiliary interferometer. However, the authors do not take into account the need to use an absolute wavelength reference.

In this work, we propose combining two reference channels of an optical frequency domain reflectometer (auxiliary interferometer and gas cell) into one channel and subsequent algorithmic decomposition of data from this channel into two informative parts. The use of empirical mode decomposition and frequency filtering is, of course, nothing new. However, in our work, for the first time to our knowledge, we consider not the processing of initial data using empirical mode decomposition, but their formation. In addition, we have not previously found works where they were used specifically in OFDR. It should also be noted that among the approaches aimed at optimizing the OFDR design, such methods, according to our information, have not yet been applied. 

## 2. Methods

### 2.1. Experimental Setup

**Figure 2:**
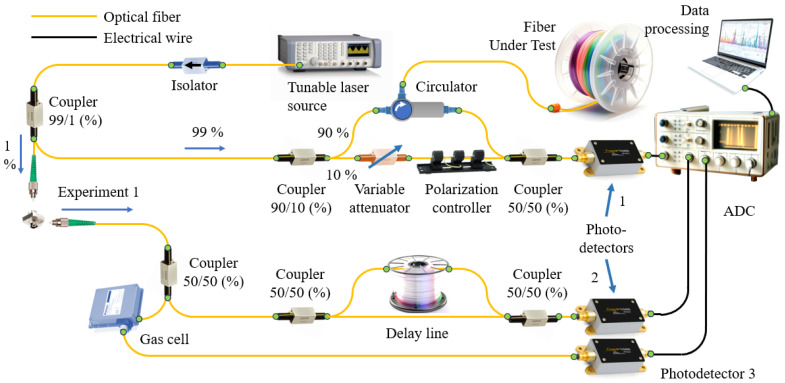
Experimental setup 1.

To conduct the experiments, two experimental setups were constructed. Let us consider the first of them (Figure 2). Like most standard optical frequency domain reflectometers, this experimental setup consists of two Mach–Zehnder interferometers and a gas cell channel. All components were placed on the table, so it was convenient for us to make modifications to the installation during the study.

**Figure 3:**
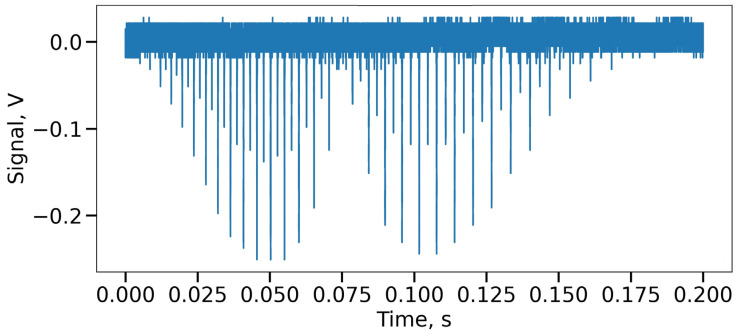
Recording of the gas cell channel in time.

The radiation from a tunable high-coherence laser source Keysight 81606A, linearly varying from 1530 to 1570 nm, was introduced through a IO-H-1550 Fiber Isolator (Thorlabs, Newton, NJ, USA) into a coupler (all couplers in the circuit are AFR SBC Series), dividing the radiation into a ratio of 99/1. The linewidth of the radiation source determines not only the maximum length of the optical fiber under study, but also the visibility of the interference pattern, on which the quality of the signal and, consequently, the accuracy of event localization depends. That is why a laser with high radiation coherence was chosen. Let us first consider the optical path that 99% of laser radiation travels. After passing the first coupler, the radiation is divided again, but in proportions 99/1. High radiation power through the optical circulator AFR FCIR1310/1550 (Advanced Fiber Resources, Zhuhai, China) enters the studied optical fiber Corning SMF28e (a little more than 50 m long) (Corning Corp., Corning, NY, USA); the second half contains a tunable attenuator and a polarization controller, which help to ensure the required intensity and the required state of polarization of the radiation in the reference arm. The first part of the radiation scattered in the fiber goes back through the circulator and interferes with the second half of the 50/50 coupler. The interference beat frequency, which carries information about the coordinate and optical properties of the medium at a given point, is sent to photodetector 1 Femto HCA-S-200 (FEMTO Messtechnik GmbH, Berlin, Germany). The electrical signal from the photodetector then goes to one of the analog-to-digital converter inputs. To visualize raw data in real time, a LeCroy WaveRunner 606Zi oscilloscope (Teledyne Technologies International Corp., Thousand Oaks, CA, USA) was selected as an ADC. The data from the oscilloscope was then sent to a desktop computer for post-processing. Now let us consider the part of the optical signal that was formed after the branching of 1% of the radiation. This radiation was also divided into two channels. The first of them was another Mach–Zehnder interferometer (auxiliary interferometer), consisting of two symmetric couplers and a delay line. Photodetector 2 (PDA05CF2, by Thorlabs, Newton, NJ, USA) recorded a signal representing the beat signal. The electrical signal coming from this detector was also sent to one of the oscilloscope channels. The second channel contained a wavelength reference (gas cell) (HCN, 50 mTorr, Wavelength References, Corvallis, OR, USA) and photodetector 3 (Thorlabs PDA05CF2). Since the laser frequency is tuned in time, the time sweep of the signal passing through the gas cell will be a set of peaks that characterize its spectrum (Figure 3). Based on the set of these peaks, it is possible to identify those points in the tuning function that correspond to a particular frequency of laser radiation at a certain moment in time.

**Figure 4:**
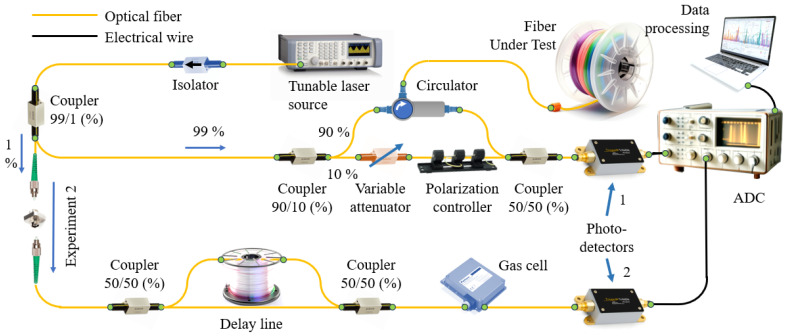
Experimental setup 2.

When describing the second experimental setup (Figure 4), it should be noted that the part of it through which 99% of the branched radiation propagates completely repeats the similar part of the first experimental setup.

**Figure 5:**
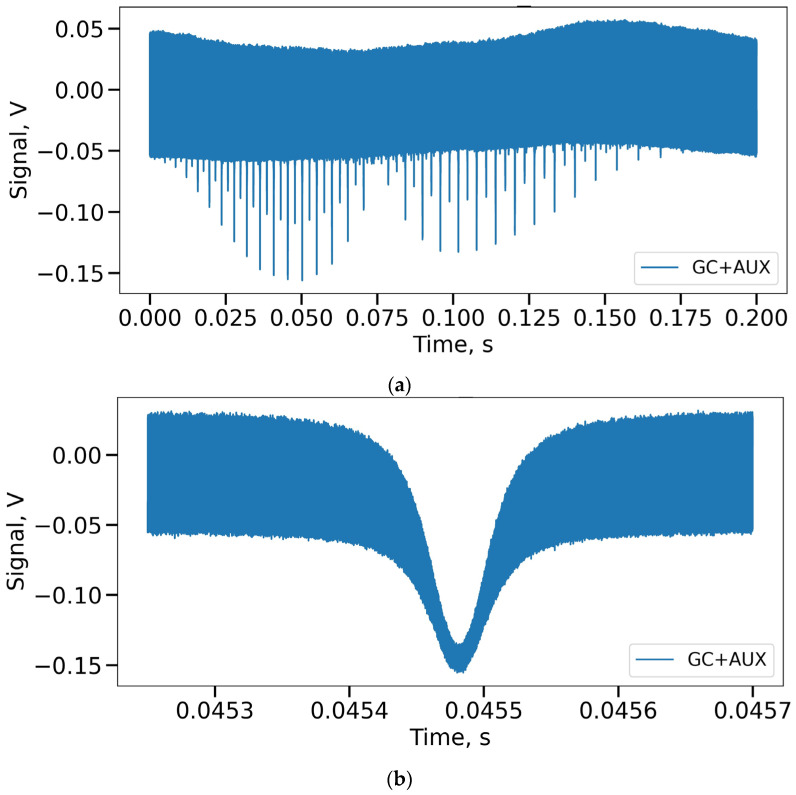
Data received from the combined reference channel of setup 2: (**a**) complete data set; (**b**) one of the gas cell peaks enlarged.

The difference lies in the additional channels, when 1% of the radiation branched by the coupler, instead of being re-divided into two equal parts, enters the auxiliary interferometer, and then follows through the gas cell to photodetector 2. This configuration avoids the use of a third photodetector and also eliminates one channel of the analog-to-digital converter. The data obtained over time by photodetector 3 is presented in Figure 5.

As can be seen from the above figure, some gas cell peaks and the frequencies of the auxiliary interferometer are visually identified in these data, but their mixing does not allow further processing: Zero Crossing (ZC) [31,32] or a more flexible algorithm—Equal Frequency Resampling (EFR) [33,34]. In addition, those peaks that characterize the beginning and end of the radiation wavelength scanning are not intense enough and are very much mixed with the signal of another channel, which makes it difficult to determine the position of their maxima. Therefore, it is necessary to apply digital signal processing techniques to separate the channels and extract useful information. The next part of this article is devoted to a description of these methods.

To conduct experiments with both setups, we used the following regimes: the power of continuous optical radiation emitted by the laser into the circuit was 10 mW. The wavelength sweeping speed was 200 nm/s. Starting wavelength: 1530 nm; final wavelength: 1570 nm. The speed of the reverse wavelength change was not controlled. The ADC sampling rate for both channels was 250 ms/s, so due to the sampling theorem, the highest frequency of the signal might be up to 125 MHz.

### 2.2. Data Processing Methods

The standard frequency filter [35] and empirical mode decomposition (EMD) [36] were chosen as data processing methods. The frequency band of the auxiliary interferometer for the used shoulder difference and laser characteristics is in the range of 8.1–8.3 MHz. A frequency of 100 kHz was empirically selected as the cutoff frequency of the filter to isolate the gas cell signal. This filter made it possible to obtain a fairly smooth gas cell signal without significant suppression of peaks. To perform the tasks posed in this study, an FIR filter of the ‘equiripple’ type was used in the MATLAB and Python environments. The attenuation coefficient in the filter suppression band (the difference in amplitudes between the remaining and filtered part of the signal) was chosen to be 60 dB.

The EMD has found its application in many fields of science and technology, including coherence reflectometry methods, but until now, only in the time domain [37,38,39]. Its essence lies in the fact that the original signal *X*(*t*) using iterative search is decomposed into independent signals, the so-called empirical modes (not to be confused with optical modes in an optical fiber):

(2) $$X\left(t\right)=\overset{N}{\underset{j=1}{\sum }}c_{j}\left(t\right)+r_{N}$$

where *c*_j_(*t*) is the empirical mode with number *j*, *r_N_* is the remainder, and *N* is the total number of modes.

At the first stage, it is necessary to find the maximum and minimum values of the signal *X*(*t*) received from photodetector 3 in a certain vicinity, that is, its local extrema. Using these local extremes and polynomial interpolation, the upper and lower envelopes of the signal are calculated—*T*(*t*) and *B*(*t*), respectively.

Next, the average value for the two envelopes is calculated element by element:

(3) $$m_{1}\left(t\right)=0.5\left[T\left(t\right)+B\left(t\right)\right]$$

as well as the difference between the original signal and its average value at each point:

(4) $$h_{1}=X\left(t\right)-m_{1}\left(t\right)$$

where 1 denotes the first empirical mode. If the function *m*_1_(*t*) has a number of local extrema that does not differ from the number of its zero values by more than one, and its average value is zero, then it is recognized as the first empirical mode. If it does not satisfy these requirements, then the calculation continues, and a new value is assigned:

(5) $$h_{11}\left(t\right)=h_{1}\left(t\right)-m_{11}\left(t\right)$$

where the second “1”-symbol in the index is the iteration number in the calculation of the first empirical mode. The calculation is repeated iteratively until the first mode is obtained at the *k*-iteration:

(6) $$c_{1}\left(t\right)=h_{1k}\left(t\right)=h_{1\left(k-1\right)}\left(t\right)-m_{1k}\left(t\right)$$

Next, the remainder is calculated $r_{1}\left(t\right):$

(7) $$r_{1}\left(t\right)=X\left(t\right)-c_{1}\left(t\right)$$

The search for the next empirical mode starts for the function:

(8) $$X\left(t\right)=r_{1}\left(t\right)$$

However, such a process can take quite a long time (especially for large data sequences, just like in this study), and the resulting empirical modes may not be realistic, that is, they may not have the real properties of the signal. The amplitudes of oscillations in the modes will tend to be constant, and the expansion itself will generally resemble Fourier analysis, which makes the EMD method meaningless [40]. In addition, this approach makes it difficult to find non-harmonic signals, which is absolutely not suitable for isolating the gas cell signal required in this work. Therefore, the calculation of intrinsic mode functions (IMFs), widely used in practice, was applied:

(9) $$c_{n}\left(t\right)=r_{n-1}\left(t\right)-r_{n}\left(t\right)$$

where *c_n_*(*t*) is the empirical mode of order *n* (IMF*n*), *r_n_*(*t*) is the average value of the envelopes at step *n*, and the remainder at this step is *r_0_*(*t*) = *X*(*t*).

The process stops when the expression for *D_k_* at iteration *k* becomes less than the specified value *D*_thr_:

(10) $$D_{k}=\overset{T}{\underset{i=0}{\sum }}\frac{{\left|c_{k-1}\left(t\right)-c_{k}\left(t\right)\right|}^{2}}{c_{k-1}^{2}\left(t\right)}$$

The one can also stop the process by independently setting the number of empirical modes.

Of course, after decomposition into empirical modes, we do not get the signal we were looking for, because these modes are determined without taking into account our task in automatic regime. Thus, decomposition of a signal containing combined data from an auxiliary interferometer and a gas cell leads to the receipt of 14 empirical modes, as well as one residual mode, which is a low-frequency oscillation and is not taken into account. A visual inspection of the obtained modes (presented below) show that the nature of their changes over time allows, to a first approximation, to assess their suitability for reconstructing the channel of an auxiliary interferometer or gas cell. Trial calculations have shown that the auxiliary interferometer is successfully restored using the first or first and second modes, and the gas cell channel is restored by summing 5–14 empirical modes. Thus, IMF numbers 3, 4, 14, and the residual are not used in signal reconstruction.

**Figure 6:**
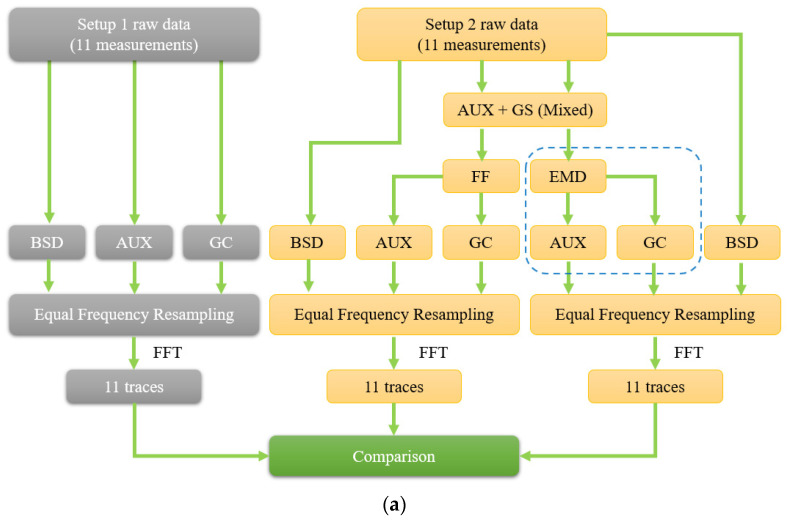

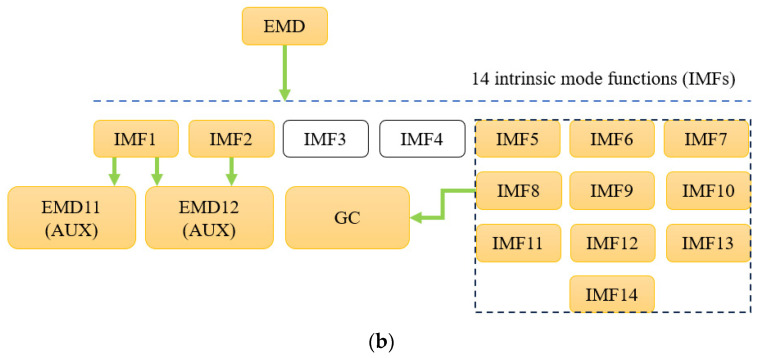
Data processing scheme: (**a**) a fragment of an algorithm with empirical modes circled in blue, excluding residual one (**b**).

The general scheme of the data processing for 11 consecutive measurements are presented in the form of an algorithm as Figure 6 shows.

Experimental setup 1 obtained backscatter data (BSD) using photodetector 1, an auxiliary interferometer signal (AUX) using photodetector 2, and a gas cell (GC) signal obtained by photodetector 3 (gray). Next, they will be processed using EFR, and thus 11 OFDR traces will be obtained, in which special attention deserves the end of the fiber optic line, where the radiation exits the optical fiber into the air. If the EFR algorithm does not work correctly, this trace location will be greatly blurred (extended by orders of magnitude). If data from the gas cell channel are processed incorrectly, the position of the Fresnel reflection at the end of the line will fluctuate quite strongly. Thus, the width of the peak at the end of the line, its height and fluctuation of the spatial position (localization error) will be used by us as the main criteria for understanding the success or failure of the method. 

Using the experimental setup 2, we received only two channels: backscattering and the auxiliary interferometer signal mixed with the gas cell signal (AUX + GC). For separation, as previously announced above, frequency filtering (FF) and empirical mode decomposition (EMD) methods were used. In the second case, the polynomial interpolation algorithms were varied. Next, in a manner similar to that described above, OFDR traces were obtained for various methods of signal decomposition with different parameters. Below, we compare both with each other and with the data obtained using experimental setup 1.

## 3. Results and Discussion

For the data obtained using setup 1, the precision error of the Fresnel reflection peak was about 0.1 mm, so it can be taken as a standard against which the effectiveness of other methods will be assessed, namely, the results of processing experimental data, obtained using setup 2, where the signals were processed by decomposition methods.

**Figure 7:**
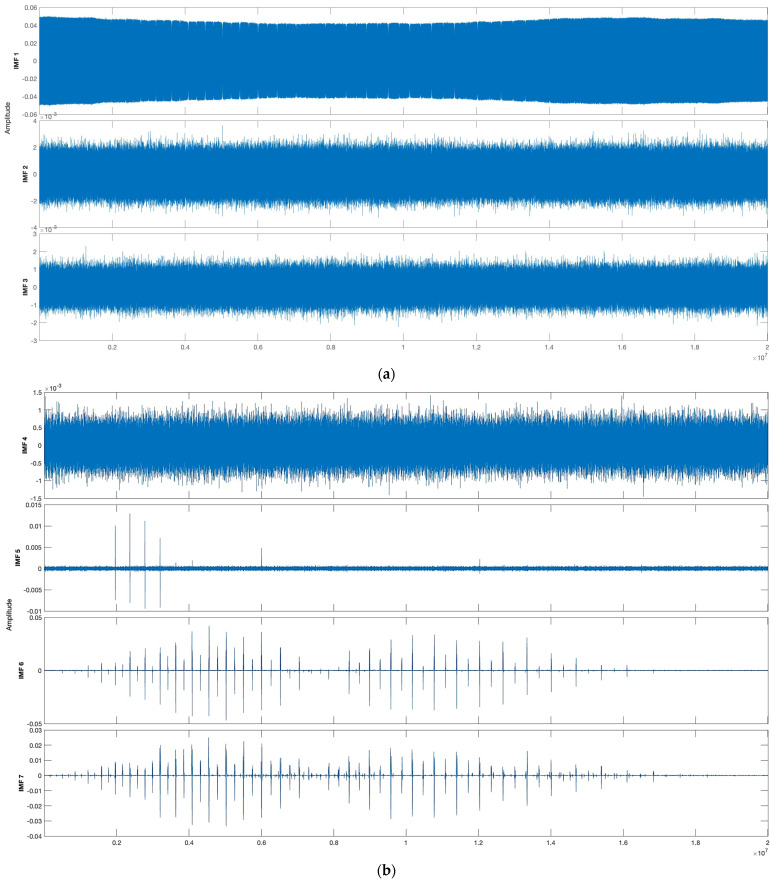

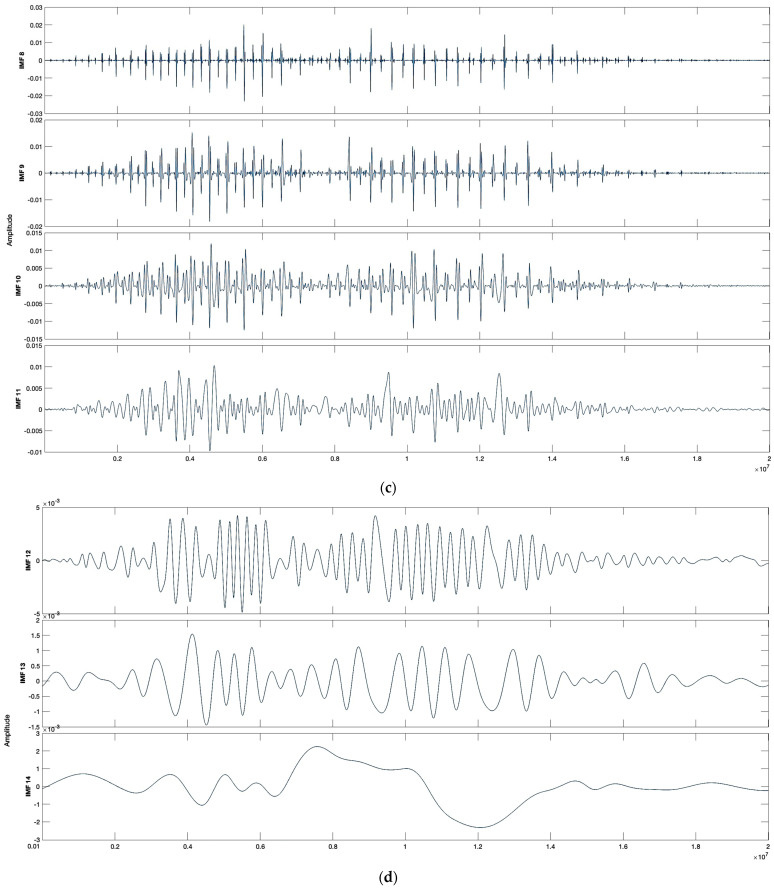
(**a**) The IMFs (1–3) of the signal. X-axis—normalized amplitude; Y-axis—time (samples). (**b**) The IMFs (4–7) of the signal. X-axis—normalized amplitude; Y-axis—time (samples). (**c**) The IMFs (8–11) of the signal. X-axis—normalized amplitude; Y-axis—time (samples). (**d**) The IMFs (12–14) of the signal. X-axis—normalized amplitude; Y-axis—time (samples).

The results of the EMD are presented in Figure 7a–d. All IMFs excluding the third, fourth, and residual one are involved in the signal reconstruction.

The following methods were chosen for comparison:

FF—separation of the channels of the auxiliary interferometer and the gas cell using digital frequency filtering using an equiripple-type finite impulse response filter;

EMD11—restoration of AUX signals of the auxiliary interferometer by the EMD using the first mode (the GC signal is formed by the IMF5-IMF14 modes);

EMD12—the same, but using the first and second empirical modes to restore AUX.

For these and the remaining methods, the following were assessed: the position of the reflecting event in fiber length, the back-reflection power (BR power), and the peak width on the trace. The first characteristic determines the correctness of using data obtained from a gas cell. Two other characteristics determine the correctness of the data coming from the auxiliary interferometer. Table 1 presents the data obtained for the FF, EMD11, and EMD22 methods.

PCHIP—channel decomposition using empirical mode decomposition, which uses the Piecewise Cubic Hermite Interpolating Polynomial to obtain the upper and lower envelopes;

woAUX—without using auxiliary interferometer data;

woGC—using auxiliary interferometer data, but without a gas cell.

The last two methods were added in order to clearly demonstrate how certain data affects the quality of traces. Visualizing each of them is unlikely to provide much valuable information, but some implementations of the experiment are still worth presenting graphically. Table 2 presents a comparison of the PCHIP, woAUX, and woGC methods.

**Figure 8:**
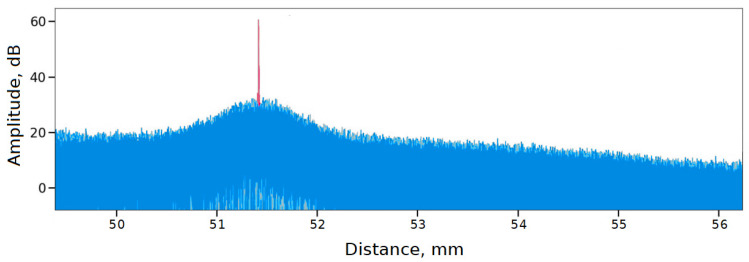
Demonstration of the need to use AUX in OFDR setup.

Figure 8 shows an implementation of the experiment for which no auxiliary interferometer data were used (blue), and also shows one trace that went through shape restoration using the EFR method based on the auxiliary interferometer data (red). It can be seen that the blue trace, due to the nonlinearity of the laser frequency tuning, does not have a clear end to the fiber line. The trace reconstructed using the auxiliary interferometer data has an order of magnitude narrower peak.

**Figure 9:**
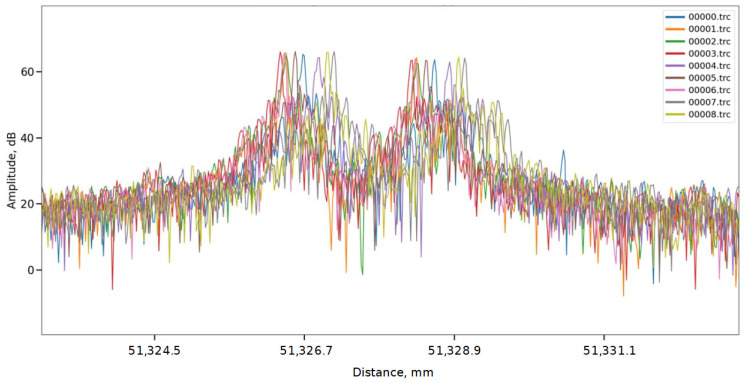
The OFDR traces reconstructed using AUX, but without the gas cell.

If you enlarge this narrow peak, you will notice that the reflection from the end consists of two peaks: the first of which corresponds to the FC/APC fiber connector, and the second to the protective cap placed on the connector. Figure 9 shows a series of such traces.

**Figure 10:**
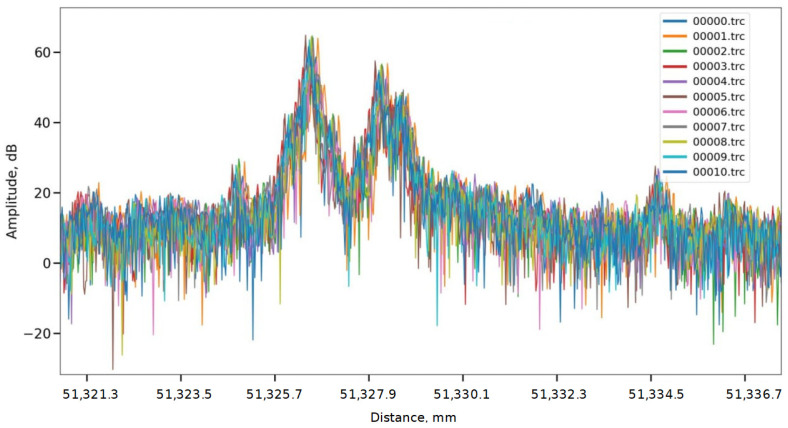
The OFDR traces reconstructed using AUX and GC (for empirical mode decomposition).

The spatial fluctuation of the peak position is on the order of 1 mm. This is due to the fact that the data presented in Figure 9 was reconstructed without using a reference wavelength—a gas cell. Figure 10 shows a different picture, where all the presented traces have the correct shape and have a much more localized back reflection at the end of the line with a fluctuation of the order of tens of micrometers.

For convenience, Table 3 summarizes all data obtained and processed according to the scheme in Figure 6 and the data presented in Table 1 and Table 2. We also added the calculation time to this table. In this table, by width and BR power, we simply mean the average value over 11 traces, and by localization error, we refer to the standard deviation given by the formula:

(11) $$\Delta =\sqrt{\frac{\sum {x_{i}}^{2}-\frac{1}{n}{\left(\sum x_{i}\right)}^{2}}{\left(n-1\right)}}$$

where *x_i_* are the results of individual coordinate measurements and *n* = 11 are the number of measurements.

The table shows that the signal intensity practically does not vary depending on the method if data from both the GC and AUX channels are used. Without the AUX channel, the intensity of the back reflection is much less. This is due to the desynchronization of the Fourier transform components caused by the nonlinearity of the optical signal frequency tuning. The same desynchronization also blurs the peak. This is why measurement without AUX produces an unrealistically long reflection event. The remaining traces, where an auxiliary interferometer is used, give the best result in terms of the length of the event. Of particular interest is the precision—the repeatability of the coordinates of the event. Unfortunately, we do not have access to an alternative method for measuring the length of the fiber under tests with comparable accuracy, so we will operate with the criterion of precision. It can be seen that all channel decomposition algorithms have shown good results. The FF method provided the best decomposition and the length accuracy was 0.106 mm (near the selected reference of 0.1 mm). The calculation using this algorithm turned out to be quite long (40 s). The reasons for this were the digital filtering settings, in particular the high filter order of 3500. In the future, we plan to conduct experiments with lowering the filter order and assessing the decomposition accuracy. Empirical mode decomposition lasted 18 s, and the degradation in accuracy compared to digital filtering was negligible. The empirical mode decomposition method, but already in the PCHIP mode, showed simultaneously the lowest accuracy and the longest calculation time for all EMD methods (20 s). To estimate the computing time, we used a laptop with an 8-core 3.5 GHz processor (4x avalanche + 4x blizzard cores), 8 GB of RAM, and 256 GB SSD.

Due to the wide spectral diversity of the signals of the gas cell and the auxiliary interferometer, it seems possible to use a digital filter with a higher cutoff frequency, cutoff slope, and lower order, which will significantly reduce signal processing time. However, in this case, high-frequency oscillations added by the filter to the gas cell signal distort the shape of its maximum and reduce the accuracy of calculating the position of the reflection from the end of the fiber.

In the case of empirical mode decomposition, the number of modes necessary to reconstruct a particular component still needs to be estimated. However, when performing this task, the EMD method stops after a small number of iterations, which significantly saves resources and therefore is more suitable for real-time measurements.

## 4. Conclusions

In this article, we present a method for reducing the cost of the hardware of an optical reflectometer in the frequency domain, which consists of replacing two reference channels, including an auxiliary interferometer and a gas cell, with one channel. To extract useful information, we used digital signal processing methods: digital frequency filtering, as well as empirical mode decomposition. We have shown that the presented approach helps to avoid the use of an unnecessary analog-to-digital converter and photodetector, while the OFDR trace is reconstructed by the EFR algorithm without loss of high resolution and with good measurement repeatability (0.11 mm—demonstrated by frequency division, FF). It should also be noted that from the pattern of back reflections constructed using the real part of the spectrum, it is possible to identify a limited number of types of events affecting the smart sensor. These events include sharp fiber bends and breaks and strong local compression, leading to an increase in the attenuation coefficient of the optical signal in the fiber. To study extremely subtle deformations and temperature changes, it is also necessary to process the imaginary part of the spectrum. We believe that these channel separation methods, which in this study have already proven their suitability for obtaining a high-quality real spectrum, will also successfully process the imaginary data. However, this statement needs to be verified; we hope to do this in our future works. We think that such modifications of OFDR will help their distribution in various smart sensor systems, including smart city systems. The use of optical frequency domain reflectometry in everyday life is hampered by the high cost of products, as well as, in some cases, the dimensions of existing frequency domain reflectometers implemented on fiber optic components. The work we provided will allow:

1. Gain additional space in the device frame or make it smaller by eliminating the detector and the analog-to-digital converter associated with it.
2. Reduce the cost of the device by using fewer components.

In addition, it seems interesting to use an apodized fiber Bragg grating instead of a gas cell, which may provide even greater benefits in reducing the cost and reducing the dimensions of the instrument in the future.

The high resolution and data acquisition speed of the optical frequency domain reflectometer allows it to be used in a variety of monitoring fields. Within the framework of the smart city concept, this is an ideal option for constant structural health monitoring of architectural monuments, as well as individual elements of buildings and structures with the transition to the “smart home” level. In seismically dangerous regions, such a device can monitor the condition of a city block after earthquakes and promptly send a signal to special services to avoid casualties. When configured to detect rapidly changing deformations [41], OFDR can act as an early warning system.

Also of interest is the use of a quasi-distributed sensor, where fiber Bragg gratings are incorporated into the line to increase the signal contrast [42,43]. This will provide a signal with a higher signal-to-noise ratio in locations where they are integrated into the optical fiber. In a smart city infrastructure, this could be any critical point, for example, a crack in a bridge or the wall of a building.

## Figures and Tables

**Table 1:** Comparison of BR power, peak width, and position for methods FF, EMD11, and EMD22.

FF			EMD11			EMD12		
Position, mm	BR Power, dB	Width, mm	Position, mm	BR Power, dB	Width, mm	Position, mm	BR Power, dB	Width, mm
51,326.1847	62.1977	1.1397	51,326.1254	62.7202	1.1016	51,326.1254	62.7217	1.1016
51,326.3167	64.1563	0.8903	51,326.2354	63.9899	0.9309	51,326.2354	63.9928	0.9309
51,326.2067	62.2726	1.1395	51,326.1914	62.2502	1.0967	51,326.1914	62.2536	1.0967
51,326.0594	64.8218	0.8906	51,326.1627	65.0560	0.8502	51,326.1627	65.0576	0.8502
51,326.1627	63.5432	0.9309	51,326.2794	63.3216	1.1411	51,326.3234	63.7181	0.9307
51,326.3014	61.9731	1.0972	51,326.2067	61.8234	1.0602	51,326.2354	61.9560	1.0975
51,326.3014	65.4454	0.8518	51,326.3167	63.4212	1.2297	51,326.3607	65.1750	0.9303
51,326.3387	62.9421	1.0993	51,325.9054	62.2549	1.0578	51,325.9054	62.2577	1.0578
51,326.3454	64.6821	0.8912	51,326.3454	64.7004	0.8913	51,326.3454	64.7022	0.8913
51,326.2794	65.0058	0.8505	51,326.1407	64.3986	0.8507	51,326.1407	64.4002	0.8507
51,326.0527	63.3924	1.0203	51,326.1254	63.0790	1.0588	51,326.1254	63.0813	1.0589

**Table 2:** Comparison of BR power, peak width, and position for PCHIP, woAUX, and woGC methods.

PCHIP			woAUX			woGC		
Position, mm	BR Power, dB	Width, mm	Position, mm	BR Power, dB	Width, mm	Position, mm	BR Power, dB	Width, mm
51,326.3827	62.0417	1.0606	50,175.8570	36.8398	2372.2929	51,222.6125	66.0418	1.4402
51,326.5654	64.2049	0.8505	50,276.2650	37.1411	2321.6339	51,230.5545	65.5999	1.3163
51,326.2067	62.2920	1.1794	50,810.6889	36.8970	3293.5291	51,236.6045	62.2696	1.6055
51,325.6567	65.0999	0.8900	51,025.1229	37.3712	3582.0933	51,235.9445	65.5210	1.3092
51,326.1914	63.8136	0.9305	51,141.3929	37.1861	3798.1979	51,239.1125	66.2181	1.3153
51,326.2067	62.1008	1.0962	51,259.1809	36.9247	4016.6688	51,241.0925	62.9372	1.9337
51,326.7194	65.1899	0.9304	51,004.4649	37.4301	3615.8351	51,223.2065	66.2919	1.2677
51,325.8987	62.9632	1.0994	50,940.7529	36.8228	3557.9447	51,226.7265	64.8821	1.4777
51,326.3674	64.2325	0.9707	50,947.9029	37.0939	3534.0229	51,228.6405	67.0267	1.2723
51,326.1407	63.7534	1.0924	50,821.9529	36.4771	3468.5312	51,221.8865	67.5454	1.1477
51,325.7734	63.3411	1.0202	50,510.8069	36.8486	2857.7540	51,233.8105	66.4518	1.3157

**Table 3:** Comparison of different channel decomposition methods.

Decomposition Method	Precision, mm	Width, mm	BR Power, dB	Computation Time, s
DF	0.106	0.982	63.676	40
EMD11	0.119	1.024	63.365	18
EMD12	0.129	0.982	63.574	18
PCHIP	0.322	1.011	63.548	20
woAUX	347.629	3310.773	37.003	N/A
woGC	6.852	1.400	65.526	N/A

## Data Availability

The experimental data presented in this study are available upon reasonable request from the corresponding author.
